# Supplementary material for: SHROOM3 Deficiency Aggravates Adriamycin-Induced Nephropathy Accompanied by Focal Adhesion Disassembly and Stress Fiber Disorganization
Source: Cells. 2025 Jun 13;14(12):895. doi: 10.3390/cells14120895 (PMC12190666; doi:10.3390/cells14120895)
Supplement: Supplementary file 1 [file cells-14-00895-s001.zip › cells-3609477-supplementary.pdf]

A B

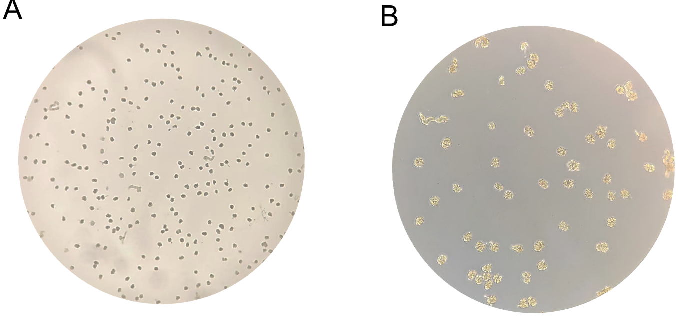

Figure S1. Representative images of isolated glomeruli. Light microscopy at objective magnification 4× (**A**) and 10× (**B**).

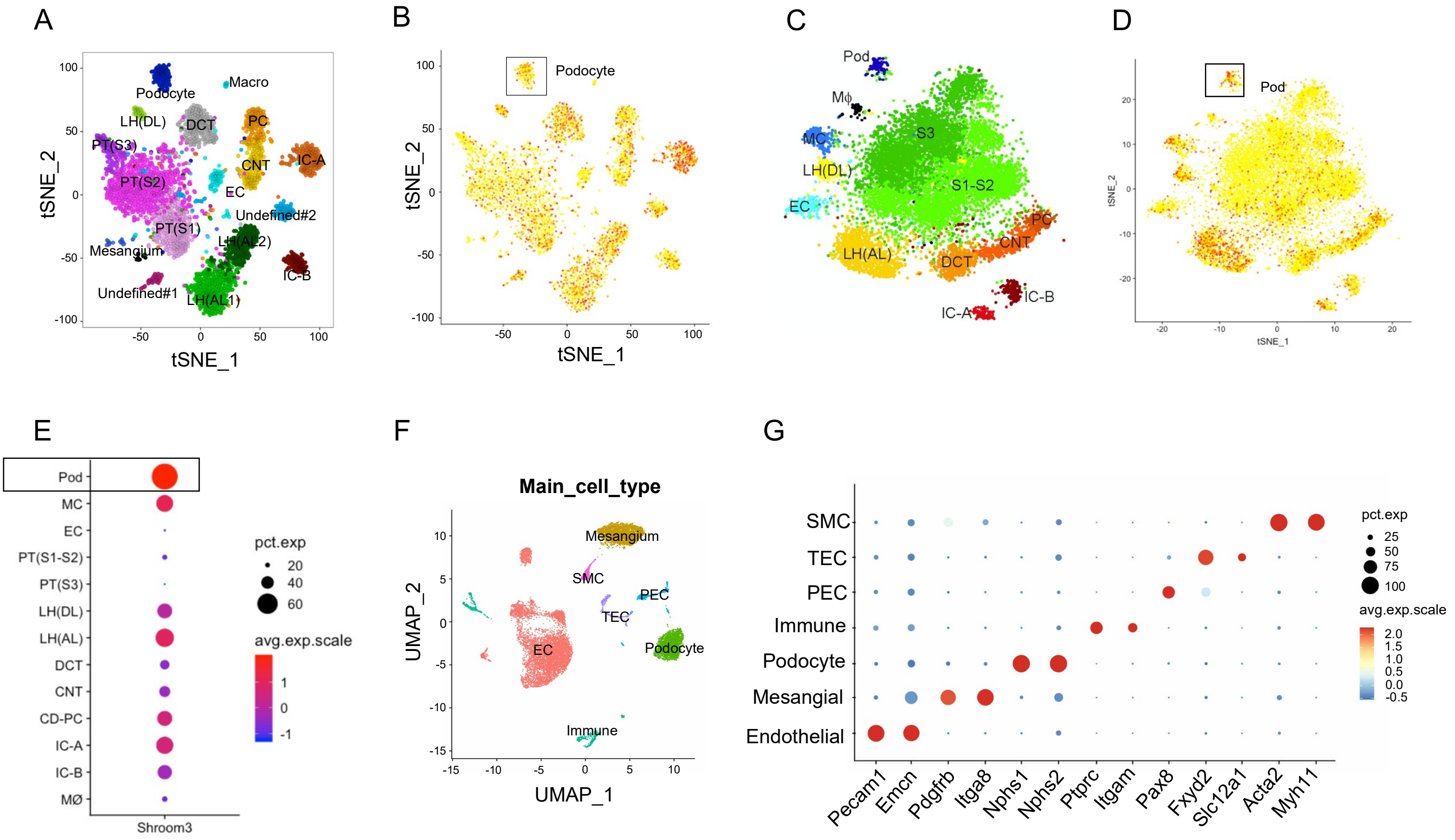

Figure S2. Single-nucleus/single-cell transcriptomic characterization of *SHROOM3* expression in adult human and mouse kidney. **(A-E)** snRNA-seq analysis from KIT database (http://humphreys-lab.com/SingleCell/search.php). (**A**) t-SNE plot of major cell populations in adult human kidney. (**B**) Human *SHROOM3* expression visualization. (**C**) t-SNE plot of major cell populations in adult mouse kidney. (**D**) Mouse *Shroom3* expression visualization. (**E**) Dot plot showing cell type-specific *Shroom3* expression in mouse kidney. (**F**,**G**) Adriamycin nephropathy dataset (GSE146912) analysis showing UMAP of dynabeads-enriched glomerular cell populations (**F**) and cell type-specific marker expression (**G**).

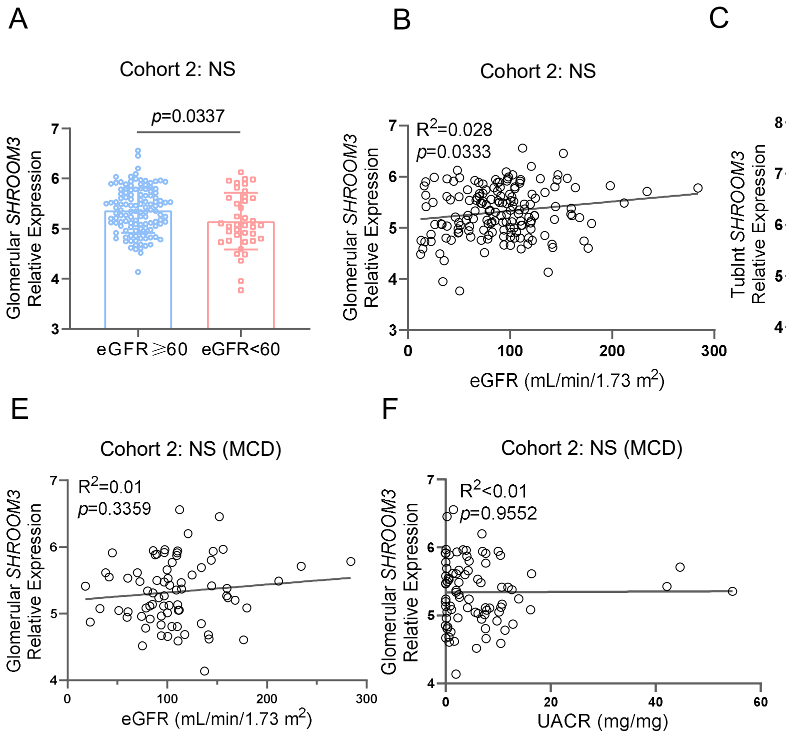

Figure S3. Expression pattern of *SHROOM3* in proteinuric kidney diseases from Nephroseq.

(**A-D**) *SHROOM3* expression patterns in nephrotic syndrome cohorts (Cohort 2 and 3). Glomerular (**A**) and tubulointerstitial (**C**) *SHROOM3* expression in patients with eGFR ≥ 60 versus <60 mL/min/1.73 m². Correlation between eGFR and SHROOM3 expression in glomerular (**B**) and tubulointerstitial (**D**) compartments. (**E&F**) Pearson linear correlation analysis of glomerular *SHROOM3* expression with eGFR (**E**) and UACR (**F**) in MCD patients from Cohort 2.

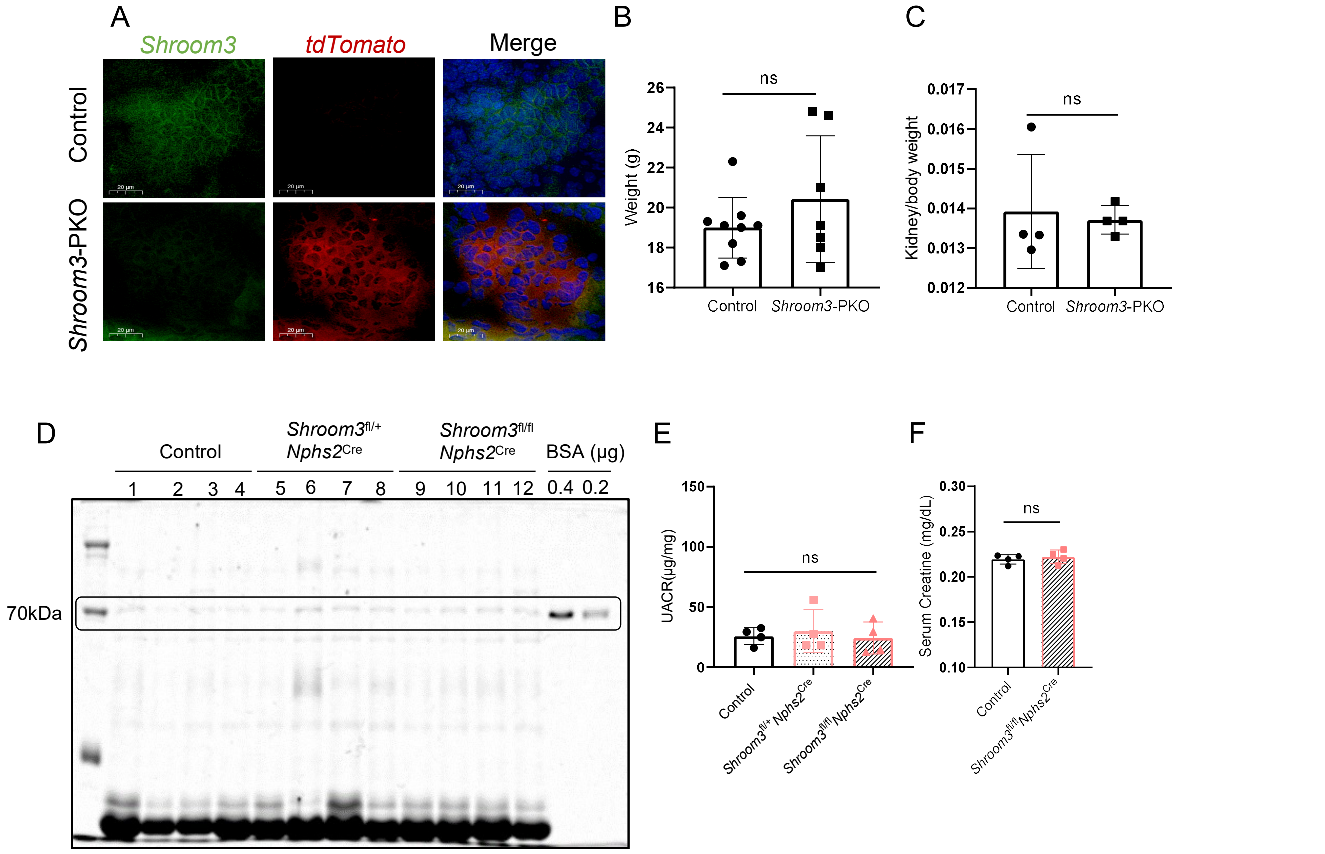

Figure S4. Characterization of podocyte-specific *Shroom3* knockout mice. (**A**) Fluorescence in situ hybridization (FISH) showing *Shroom3* mRNA (green) and tdTomato reporter (red) expression in kidney sections of newborn mice. Scale bar: 20 µm. (**B&C**) Analysis of 3-month-old mice showing (**B**) body weight and (**C**) kidney-to-body weight ratio in *Shroom3*-PKO and littermate controls. (**D**) Representative Coomassie Brilliant Blue-stained SDS-PAGE of urinary proteins from control (lanes 1-4), *Shroom3*$^{fl/+}$;*Nphs2*^{Cre} (lanes 5-8) and *Shroom3*$^{fl/fl}$;*Nphs2*^{Cre} (lanes 9-12) mice, with BSA standards (0.4, 0.2 µg). (**E**) Quantification of urinary UACR in control, *Shroom3*$^{fl/+}$;*Nphs2*^{Cre} and *Shroom3*$^{fl/fl}$;*Nphs2*^{Cre} mice. (**F**) Quantification of Serum creatinine in control and *Shroom3*$^{fl/fl}$;*Nphs2*^{Cre} mice.

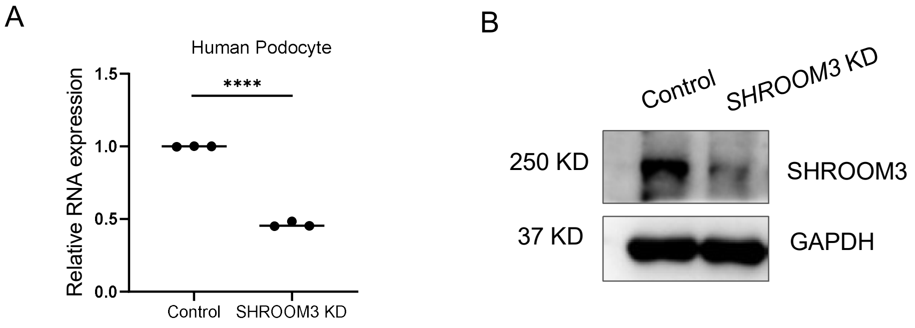

Figure S5. Validation of *SHROOM3* knockdown in human podocytes. (**A**) RT-qPCR quantification of *SHROOM3* mRNA expression in control and *SHROOM3* KD human podocytes. Unpaired Student's t-test, ****p < 0.0001. (**B**) Representative immunoblot showing SHROOM3 protein levels in control and *SHROOM3* KD podocytes, with GAPDH as loading control.

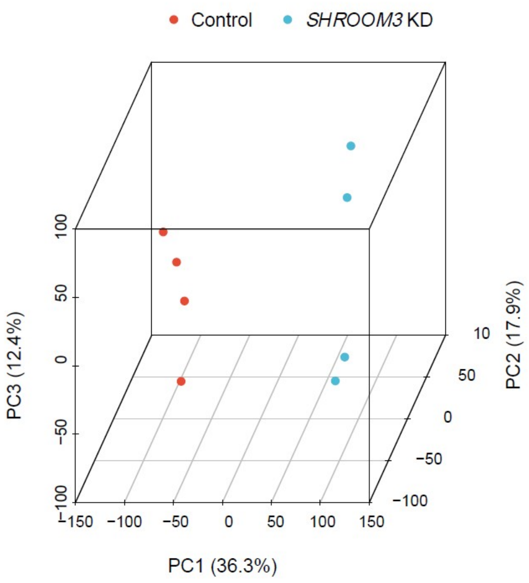

Figure S6. Global transcriptomic changes in *SHROOM3*-deficient podocytes. Three-dimensional principal component analysis (PCA) of RNA-sequencing data from control (red) and *SHROOM3* KD (blue) human podocytes, showing distinct transcriptional profiles. The percentages in parentheses indicate the variance explained by each principal component.

Supplementary Table S1. Oligonucleotide sequences used for RT- qPCR analysis and genotyping in mouse models.

Gene	Primer
**Human qPCR**	
SHROOM3	F: 5'-CCCTCTCGGGGCGTCTAGCC-3'
	R: 5'-GCCCAGCACTACTCGCTCCC-3'
GAPDH	F: 5'-TGTTGCCATCAATGACCCCTT-3'
	R: 5'-CTCCACGACGTACTCAGCG-3'
**Mouse genotying**	
Shroom3-loxp	F: 5'-CCCACAAACAAATCGTCCCTACTA-3'
	R: 5'-TGTAACTGACAATTTGCACAGGC-3'
Nphs2-Cre	F: 5'-GCGCTGCTGCTCCAG-3'
	R: 5'-CGGTTATTCAACTTGCACCA-3'
tdTomato-wt	F: 5'-AAGGGAGCTGCAGTGGAGTA-3'
	R: 5'-CCGAAAATCTGTGGGAAGTC-3'
tdTomato-mut	F: 5'-CTGTTCCTGTACGGCATGG-3'
	R: 5'-GGCATTAAAGCAGCGTATCC-3'
**Mouse qPCR**	
Shroom3	F: 5'-GCGTGTTCCAATCACCACAG-3'
	R: 5'-AGCTCCACGTTGCTTTGTCT-3'
Gapdh	F: 5'-TGAGCAAGAGAGGCCCTATC-3'
	R: 5'-AGGCCCCTCCTGTTATTATG-3'
Nphs1	F: 5'-ATGGGAGCTAAGGAAGCCACA-3'
	R: 5'-GATGGAGAGGATTACGCTGGG-3'
Nphs2	F: 5'-GACCAGAGGAAGGCATCAAGC-3'
	R: 5'-GCACAACCTTTATGCAGAACCAG-3'
Podxl	F: 5'-CAGCAGCTCTGGCTCCACGC-3'
	R: 5'- TGTCTGGCTGGTGAGGGGCT-3'
Synpo	F: 5'-CTCAGTGACTCTGATTCCAG-3'
	R: 5'-TGTGCCTCATCTAACTCCAG-3'

Supplementary Table S2. Short hairpin RNA sequences used for *SHROOM3* knockdown and control in podocyte cell cultures.

RNAi-Primer	5'-sequence-3'
shSHRM-3'utr	CCGGGTCTGCAACATAAAGCCTTAACTCGAGTTAAGGCTTTATG TTGCAGACTTTTTG
shcontrol	CCGGGATCGTACTAGCTATGCAACTCGAGTTGCATAGCTAGTAC GATCTTTTTG
